# Effects of physiotherapy on breathing cycle after thoracic surgery measured with impedance pneumography in a prospective clinical comparison

**DOI:** 10.1007/s11748-025-02199-y

**Published:** 2025-09-26

**Authors:** Sabina Lähteenmäki, Milla Juutinen, Jari Viik, Heidi Mahrberg, Jari Laurikka

**Affiliations:** 1 Faculty of Medicine and Health Technology, Tampere University, Tampere, Finland; 2 Tampere University Heart Hospital, Tampere, Finland

**Keywords:** Impedance pneumography, Thoracic surgery, Rehabilitation

## Abstract

**Objectives:**

Impedance pneumography (IP) records respiratory cycle and provides non-invasive means to evaluate changes after thoracic surgery. This comparative study evaluated if changes after thoracic surgery can be modified by two pulmonary rehabilitation modalities.

**Methods:**

88 patients undergoing thoracic surgery were randomly allocated to either positive expiratory pressure (PEP) or inspiratory muscle training (IMT) physiotherapy group. Physiotherapy was performed and IP recorded preoperatively and at first and second postoperative days (POD1 and POD2) during tidal breathing. Full three timepoint IP data were collected from 81 patients (42 in the PEP group, 39 in the IMT group). Average inspiration and expiration time in seconds (TinspAvg and TexpAvg) and average breathing frequencies (BrthFreqAvg) were calculated from a 10-min measurement period and assessed as primary outcomes. Results were also assessed in blocks of different surgical techniques (thoracotomy or VATS) and the extent of surgery (pulmonary resection or minor thoracic operation).

**Results:**

TinspAvg and TexpAvg decreased after surgery but started to increase in the IMT group between POD1 and POD2 with no significant difference compared to PEP group. Among patients operated with thoracotomy TexpAvg was slightly but insignificantly higher in the IMT group at POD2. The ratio between the time of expiration and the time of inspiration (EI ratio) was significantly higher in the IMT group with thoracotomy (difference between groups over three timepoints, (*p* = 0.044) and at POD1 (*p* = 0.015)).

**Conclusions:**

IMT seemed to enhance expiration specifically among thoracotomy patients and thus may provide means for enhancing the recovery after thoracic operations.

**Clinical trial registration:**

NCT02931617/U.S. National Library of Medicine, ClinicalTrials.gov.

## Introduction

Respiratory function may be impaired after thoracic surgery due to wound pain, impaired diaphragmatic function, or reduced ventilated volume. Impaired respiration after lung surgery predisposes to postoperative pulmonary complications (PPCs), hinders recovery, and leads to longer duration and higher overall cost of hospitalization [1–3]. Recovery is promoted by respiratory physiotherapy, which benefits have been demonstrated after major lung resections [4–12]. Early pre- and postoperative rehabilitation in resective lung surgery is advised by European Respiratory Society/European Society of Thoracic Surgeons (ERS/ESTS) guidelines (level of evidence 2 +, grade of recommendation C) [13].

Benefits of inspiratory muscle training (IMT) for pre- and postoperative rehabilitation has been demonstrated by several studies, which indicate decreased incidence of PPCs and shorter hospitalization in surgical patients [9, 14–16]. Our two previous studies demonstrated IMT being as efficient as water bottle positive expiratory pressure (PEP) training among minor lung operation patients [17] as well as major lung resection patients, and as a more feasible form of physiotherapy in cases with postoperative air leakage [18].

Spirometry and pneumotachography (PNT) are often referred to as golden standards in the measurements of pulmonary functions [19]. However, spirometry results depend on the patient’s ability and performing, and it is also contraindicated for patients with air leak after thoracic surgery. Impedance pneumography (IP) measures respiration noninvasively using bioimpedance of the thorax [20]. IP is currently in clinical use in respiratory rate monitoring and in pediatric asthma diagnostics, but other applications are still under investigation and, therefore, not yet in wide clinical practice, e.g., in thoracic surgery. IP enables evaluation of respiration not requiring exertion or cooperation and thus provides a promising method for postoperative evaluation of physiotherapy treatment and overall recovery.

The main hypothesis of the study was that IMT physiotherapy could promote longer inspiration in tidal breathing after physiotherapy session and, therefore, lower breathing frequency. We evaluated the effects of respiratory physiotherapy in breathing cycle using IP measurements. The null hypothesis was that there is no difference between IMT and PEP physiotherapy in breathing values measured by IP over three timepoints.

## Study design and methods

Study population consisted of 88 patients who underwent either lung resection surgery or lung biopsies and other minor thoracic operations at Tampere University Heart Hospital, Finland, between May 2013 and February 2016. The sample size was calculated based on observed differences in FEV1 values from a pilot study involving 38 patients. The number of patients needed to detect this difference was calculated to be minimum of 17 patients in both treatment groups. Study patients have been described in detail in our two previous works [17, 18]. Patients unable to co-operate because of a psychiatric condition, neurological ailment severely affecting respiration, intoxication upon admission, severe interstitial pulmonary disease, infective pulmonary disease or contagious infection including tuberculosis, recent or acute febrile respiratory infection, preoperative cardiac pacemaker, severe respiratory failure, or legally incapacitated patients were excluded from the study.

Participants were randomly allocated into two treatment groups after signing the informed consent files. Recruiting was terminated upon enrolment of the predetermined number of patients as reported in our previous studies [17, 18]. Patients were randomized (with computerized random numbers in sealed envelopes) 1:1 to parallel open label groups receiving PEP physiotherapy (44 patients) or IMT (44 patients) prior to surgery. The randomization was in two blocks depending on the anticipated resection (resective surgery or minor thoracic surgery) and the surgical technique was decided by operative surgeon. No conversions of planned surgical techniques occurred.

The study protocol (R13037) was approved by the Ethics Committee of the Pirkanmaa Hospital District, Tampere, Finland, and was conducted in accordance with the Declaration of Helsinki (as revised in 2013).

Dedicated physiotherapist (HM) instructed the assigned physiotherapy (PEP or IMT) to each patient on the preoperative day (Preop), and on the first (POD1) and second postoperative day (POD2). Exercises were performed once a day with the physiotherapist, and the patients were encouraged to do independent exercises at least five times a day. In the water bottle PEP group, a basic pressure of 10 H2Ocm was used. Patients were provided with a bottle with thick blowing tube, and the bottle was filled with water to provide pressure of 10 H2Ocm. Patients were instructed by the same dedicated physiotherapist to give long steady blows through the tube into the water-filled bottle. A single exercise consisted of ten consecutive blows. The water level and hence the expiratory resistance was lowered to 5–7 H2Ocm if air leakage increased to 200 ml or more during exercise. For IMT physiotherapy, a ThresholdIMT (Philips Respironics, Murrysville, Pennsylvania, United States) apparatus was employed. Pressure was set at 20% of the individuals´ preoperative maximal inspiratory pressure (MIP). Patients were instructed by the same physiotherapist to take deep steady inhalations through the ThresholdIMT apparatus, and a single exercise consisted of ten concecutive inhalations. The pressure was kept the same throughout the follow-up period. Furthermore, patients were instructed to walk at least 3–4 times at the first postoperative day and at least five times at the second postoperative day. Patients kept diaries about exercises done independently and their compliance in performing the exercises were recorded. Compliance was excellent in the study population throughout the follow-up and there were no differences between treatment groups in compliance.

Pulmonary function tests were conducted on all three days of the study: preoperatively before allocation, and on POD1 and POD2 after performing the physiotherapeutic exercises. After Pulmonary function testing, the patient was doing tidal breathing in a recumbent position for 10 min and bioimpedance data were recorded with a small custom-designed recording device (Tampere University of Technology, Finland) [21]. Two skin electrodes were placed on the sides of the thorax and other two in the arms close to the armpit [22]. PNT was used as a reference method and the signals of IP and PNT were recorded simultaneously for 1-min period before the beginning of a 10-min period of tidal breathing IP measurements. In this study, parameters were calculated as mean values of 10-min recording period. IP and PNT signals were processed and analyzed with Matlab R2016.

Average inspiration (TinspAvg) and expiration (TexpAvg) times and breathing frequency (BrthFreqAvg) calculated from a 10-min period and ratio between average expiration and inspiration times (EI ratio) were assessed as primary outcomes. Among seven patients, the IP measurement data had corrupted signal (which was dealt as missing data) leading to analysis of total of 81 patients results.

Statistical comparisons were made with IBM SPSS Statistics for Windows 26/28 (IBM Corp, Armonk, New York, United States). Differences between intervention groups in IP values as a function of time were analyzed using two-way mixed factors repeated measures variance analysis. Comparison of the mean values of the variables was done with the Student’s *t* test of independent variables. Differences between different timepoints within therapy group were tested with related samples Wilcoxon signed-rank test. Differences between the classified variables were tested by cross-tabulation and Pearson’s *χ*^*2*^ test. Differences were considered statistically significant when p < 0.05.

## Results

Patient demographics and general data are given in Table 1. There were no significant differences between intervention groups in demographics except type 2 diabetes mellitus, which was more common in the PEP group (*p* = 0.026). PEP group patients had slightly higher average NRS scores for pain than IMT group patients in POD1 among VATS operated patients or minor resection patients (VATS: 4,1 vs. 2,3; *p* = 0,01; Minor thoracic operation: 3,7 vs. 2,3; *p* = 0,02, respectively). There was no difference between intervention groups at POD2. Numeric Rating Scale (NRS) scores more than 5 were rare in both intervention groups. There were no significant differences in modalities of pain medication between intervention groups.

**Table 1:** Main parameters of the study patients in the intervention groups

Characteristics		PEP group (*N* = 42)		IMT group (*N* = 39)	*P* value		
Age, years, median (IQR)		67 (15)		66 (15)	0.463		
BMI, kg/m^2^, median (IQR)		27.0 (5.3)		27.0 (9.0)	0.668		
Sex, n (%)					0.236		
Male		27 (64%)		20 (51%)			
Female		15 (36%)		19 (49%)			
Smoking history					0.277		
Current smoker		9 (21%)		12 (31%)			
Ex-smoker		23 (55%)		13 (33%)			
Lifetime non-smoker		9 (21%)		12 (31%)			
Main diagnosis, n (%)					0.790		
Primary lung cancer (C34)		18 (43%)		20 (51%)			
Lung metastasis (C78)		10 (24%)		6 (15%)			
Benign tumors of trachea, bronchi or lung (D14, D21)		4 (10%)		3 (8%)			
Nondefined Lung tumor (D37, D38)		5 (12%)		3 (8%)			
Pleural fluid		1 (2%)		1 (3%)			
Pneumothorax		0		1 (3%)			
Pleuritis		0		1 (3%)			
Suspected interstitial pulmonary disease		1 (2%)		0			
Non-defined lung tumor or other abnormal imaging of the lung		3 (7%)		4 (10%)			
Co-morbidities, n (%)							
COPD		10 (24%)		8 (21%)	0.721		
Asthma		3 (7%)		5 (13%)	0.392		
Asbestosis		5 (12%)		3 (8%)	0.525		
Coronary artery disease		4 (10%)		2 (5%)	0.450		
Hypertension		20 (48%)		17 (44%)	0.716		
Type II diabetes		10 (24%)		2 (5%)	**0.018**		
Type of surgery, n (%)					0.853		
Thoracotomy		**17 (40%)**		**15 (38%)**			
Lobectomy		8		9			
Bi-lobectomy		1		0			
Biopsy/excisio		7		3			
Wedge resection		1		2			
Pneumonectomy		0		1			
VATS		**25 (60%)**		**24 (62%)**			
Lobectomy		9		11			
Bi-lobectomy		1		0			
Biopsy/excisio		12		8			
Wedge resection		3		4			
Pleurectomy		0		1			

The type of surgery was thoracotomy (PEP: *n* = 17; IMT: *n* = 15) or video assisted thoracic surgery (VATS) (PEP: *n* = 25; IMT: *n* = 24). The most common procedures were lobectomies, bi-lobectomies, biopsies or small excision, or wedge resections (Table 1). Pulmonary resective surgery was done just as likely by thoracotomy (*n* = 21) than VATS technique (*n* = 21), but VATS technique was more often chosen for minor thoracic operations (VATS = 32, thoracotomy = 14, respectively).

Full three timepoint IP data were successfully recorded from 81 patients (42 in the PEP group, 39 in the IMT group).

**Fig. 1:**
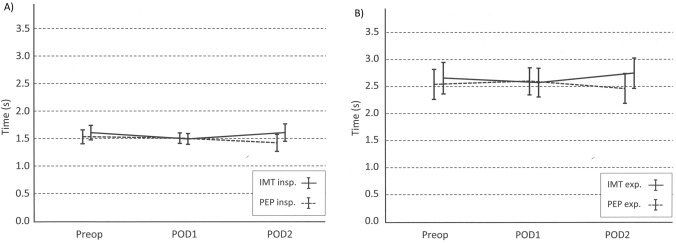
Trends in the inspiration time (**a**) and expiration time (**b**) with CI at three timepoints of the study. Dotted lines illustrate PEP group, solid lines IMT group

Inspiration time (TinspAvg) dropped in both intervention groups after surgery. The decrease continued within PEP group patients, but TinspAvg increased at the level of Preop day value at POD2 in IMT group (Fig. 1a). The changes were similar regardless of the surgical technique (thoracotomy or VATS) or the amount of resected lung tissue (pulmonary resection or minor thoracic operation). There was no statistically significant difference in TinspAvg between intervention groups when variances over three timepoints were compared or at each timepoint separately, although the difference between intervention groups seemed to be increasing at POD2. (Tables 2 and 3).

**Table 2:** Respiratory cycle time parameters pre- and postoperatively in IMT and PEP group, and *p* values of repeated measures analysis of variance

	All patients			Thoracotomy			VATS		
Variable	PEP (*N* = 42)	IMT (*N* = 39)	P value	PEP (*N* = 17)	IMT (*N* = 1*5*)	*P* value	PEP (*N* = 25)	IMT (*N* = 2*4*)	*P* value
TinspAvg^a^ Pre^e^, sec (SD)	1.53 (0.40)	1.61 (0.42)	0.297	1.49 (0.29)	1.58 (0.35)	0.617	1.56 (0.46)	1.62 (0.47)	0.359
TinspAvg POD1^f^, sec (SD)	1.50 (0.33)	1.49 (0.28)		1.45 (0.28)	1.40 (0.26)		1.54 (0.37)	1.55 (0.28)	
TinspAvg POD2^g^, sec (SD)	1.42 (0.30)	1.61 (0.65)		1.42 (0.31)	1.49 (0.21)		1.42 (0.30)	1.67 (0.83)	
TexpAvg^b^ Pre, sec (SD)	2.54 (0.84)	2.66 (0.96)	0.425	2.42 (0.95)	2.71 (0.97)	0.117	2.62 (0.76)	2.62 (0.97)	0.809
TexpAvg POD1, sec (SD)	2.60 (0.99)	2.58 (0.62)		2.31 (0.64)	2.67 (0.85)		2.80 (1.14)	2.51 (0.38)	
TexpAvg POD2, sec (SD)	2.46 (0.68)	2.75 (1.06)		2.31 (0.73)	2.81 (0.95)		2.56 (0.64)	2.70 (1.15)	
BrthFreqAvg^c^ Pre, BPM (SD)	16.47 (3.61)	16.14 (4.15)	0.430	17.37 (3.87)	15.79 (3.48)	0.224	15.87 (3.37)	16.38 (4.62)	0.983
BrthFreqAvg POD1, BPM (SD)	16.30 (3.90)	16.17 (3.05)		17.32 (3.94)	16.91 (3.89)		15.60 (3.79)	15.65 (2.24)	
BrthFreqAvg POD2, BPM (SD)	16.92 (3.81)	15.73 (3.79)		17.50 (3.88)	15.48 (2.56)		16.52 (3.78)	15.90 (4.50)	
EI ratio^d^ pre (SD)	1.66 (0.36)	1.66 (0.38)	0.938	1.60 (0.39)	*1.70 (0.46)*	*0.044*	1.71 (0.33)	*1.63 (0.32)*	*0.072*
EI ratio POD1 (SD)	*1.72* (0.43)	1.75 (0.35)		1.58 (0.29)	*1.91 (0.42)*		1.81 (0.49)	*1.64 (0.26)*	
EI ratio POD2 (SD)	1.75 (0.41)	1.74 (0.41)		1.63 (0.39)	1*.89* (0.50)		1.83 (0.41)	*1.64 (0.30)*	

^a^ Mean inspiration time in seconds and standard deviation

^b^ Mean expiration time in seconds and standard deviation

^c^ Mean breathing frequency and standard deviation

^d^ Mean ratio between expiration and inspiration, and standard deviation

^e^ preoperative day

^f^ first postoperative day

^g^ second postoperative day

**Table 3:** Respiratory cycle time parameters pre- and postoperatively in IMT and PEP group among pulmonary resection and minor thoracic operation patients separately, and *p* values of repeated measures analysis of variance

	Minor thoracic operation			Pulmonary resection		
Variable	PEP (*N* = 23)	IMT (*N* = 18)	P value	PEP (*n* = 19)	IMT (*N* = 21)	*P* value
InspAvg^a^ Pre^e^, sec (SD)	1.61 (0.47)	1.66 (0.51)	0.39	1.44 (0.27)	1.56 (0.34)	0.29
InspAvg POD1^f^, sec (SD)	1.58 (0.40)	1.57 (0.28)		1.42 (0.21)	1.42 (0.26)	
InspAvg POD2^g^, sec (SD)	1.47 (0.33)	1.78 (0.90)		1.36 (0.26)	1.46 (0.24)	
ExpAvg^b^ Pre, sec (SD)	2.66 (0.89)	2.58 (0.96)	0.75	2.40 (0.77)	2.72 (0.98)	0.29
ExpAvg POD1, sec (SD)	2.74 (1.17)	2.62 (0.52)		2.44 (0.72)	2.54 (0.70)	
ExpAvg POD2, sec (SD)	2.50 (0.66)	2.95 (1.40)		2.41 (0.72)	2.57 (0.63)	
BrthFreq^c^Avg Pre, BPM (SD)	15.64 (3.70)	16.21 (4.97)	0.79	17.48 (3.33)	16.09 (3.42)	0.24
BrthFreqAvg POD1, BPM (SD)	15.64 (4.21)	15.34 (2.56)		17.09 (3.42)	16.88 (3.31)	
BrthFreqAvg POD2, BPM (SD)	16.34 (3.55)	15.19 (4.42)		17.62 (4.08)	16.19 (3.20)	
EI ratio^d^ pre (SD)	1.66 (0.32)	1.56 (0.32)	0.55	1.67 (0.41)	1.74 (0.41)	0.57
EI ratio POD1 (SD)	1.72 (0.44)	1.68 (0.29)		1.72 (0.44)	1.80 (0.39)	
EI ratio POD2 (SD)	1.73 (0.43)	1.70 (0.49)		1.77 (0.40)	1.77 (0.32)	

_a_ Mean inspiration time in seconds and standard deviation

^b^ Mean expiration time in seconds and standard deviation

^c^ Mean breathing frequency and standard deviation

^d^ Mean ratio between expiration and inspiration, and standard deviation

^e^ preoperative day

^f^ first postoperative day

^g^ second postoperative day

However, the decrease of TinspAvg between POD1 and POD2 in PEP group was statistically significant (*p* = 0.013), but the increase of TinspAvg in IMT group between POD1 and POD2 was not (*p* = 0.397). The decrease between Preop and POD1 was not statistically significant in either group (PEP: *p* = 0.554; IMT: *p* = 0.080).

Time of expiration (TexpAvg) increased in the PEP group at POD1 but decreased in the IMT group. At POD2 time of expiration increased at IMT group but decreased at PEP group (Fig. 1b) when evaluating the whole study population. The changes were similar regardless of surgical technique (thoracotomy or VATS) or the amount of resected lung tissue (pulmonary resection or minor thoracic operation). There was no statistically significant difference between intervention groups when the groups were compared in the repeated measures analysis of variance over three timepoints (Table 2). The changes in TexpAvg between Preop day and POD1 or POD1 and POD2 were not statistically significant either.

When the expiration to inspiration ratio was analyzed using the repeated measures analysis of variance, no statistically significant differences in the EI ratios between the intervention groups were noted over the studied three timepoints (Table 2). However, when comparing therapies in respect to surgical modalities, the difference in the EI ratio between the therapy groups was statistically significant among thoracotomy patients (*p* = 0.044) but not among VATS patients (*p* = 0.072) (Table 2). Among the thoracotomy patients there were statistically significant differences between intervention groups in EI ratio at POD1 (PEP = 1.58, IMT = 1.91, respectively; *p* = 0.015) but not at POD2 (PEP = 1.63, IMT = 1.89, respectively; *p* = 0.108).

When comparison was made within the original allocation groups by the extent of tissue resected (lobectomy or biopsy), there was no statistically significant difference between intervention groups (Table 3).

Breathing frequency stayed near the preoperative value at POD1 in the IMT group but decreased in the PEP group when evaluating the whole study population. At POD2 breathing frequency increased at PEP group but decreased below preoperative value at IMT group, but there was no statistically significant difference between intervention groups in the variances over three timepoints (Table 2). Among VATS patients BrthFreqAvg decreased at POD1 in both intervention groups but increased at POD2, still staying below preoperative value at IMT group. Also, among minor thoracic operation patients the BrthFreqAvg was decreasing at POD1 and POD2, but there was no statistically significant difference between intervention groups in variances over three timepoints or between medians at different timepoints.

To compensate for potential effects of non-normal distributions in the data, data logarithmic transformations were compared at different timepoints using the repeated measures analysis. However, the results remained the same. To ensure that some individual differences in the preoperative values would not affect the results, we also calculated individual ratios of each variables (postoperative values compared to same patients’ preoperative value), indicating the individual relative postoperative change in comparison with preoperative value. We used these ratios for comparison in each timepoint separately and in repeated measures analysis, but this did not change the results.

## Discussion

The benefits of IMT physiotherapy have been demonstrated by several studies, but it is not known how training may affect the different times of the respiratory cycle. In this preliminary study we compared two physiotherapy modalities, PEP and IMT, among thoracic surgery patients using impedance pneumography measurements. We aimed to investigate if IMT physiotherapy has benefits over PEP physiotherapy in immediate recovery of inspiration or expiration after thoracic surgery using impedance-based analysis of respiration.

Since PEP training involves long expirations against pressure, we assumed that it may promote longer expiration in tidal breathing after training as well. IMT physiotherapy, in comparison, uses pressure in inspiration and this promotes for long inspirations during training. Theoretically this could lead to longer inspiration times in tidal breathing after training with IMT. Since breathing consists of inspiration and expiration, decrease or increase in duration of either of them may affect breathing frequency and even circulatory physiology in postoperative patients.

Thoracic surgery may weaken lung function and affect respiratory cycle by many mechanisms. Pain and stiffening of the thoracic wall may impair both inspiration and expiration. Thoracotomy involves larger incision and in addition spreading of adjacent ribs, which often causes significant postoperative pain. Postoperative pain is less significant after VATS operation, although still present [23]. We observed slightly larger average NRS scores in PEP group when comparing VATS operated patients and in minor thoracic operation patients, but the average scores were still relatively low in both groups, and there were no observed differences in compliance doing the exercises. Therefore, this is unlikely a clinically significant difference nor affecting our results. There were no statistically significant differences in the NRS scores between treatment groups operated by thoracotomy or having a major pulmonary resection done.

Atelectasis may remain in the operated lung which may decrease the vital capacity after surgery. Breathing against atelectatic area to open the alveoli demands pressure but how this affects breathing cycle is not well-known. In addition, barotrauma to contralateral lung in single lung ventilation may occur, and the operated lung may have been traumatized by manipulation in operation and subsequent inflammation.

The pulmonary resection patients (e.g., lobectomy, bi-lobectomy, and pneumonectomy) and patients with other minor thoracic operation (e.g., pulmonary biopsies) as groups differ from each other by the effects the surgery has on patients’ respiration. In pulmonary resection the ventilatory volume is decreased by the volume of the resected lobe(s) and the ventilatory capacity is decreased altogether, but this volume may be replaced to some degree if the adjacent lobe(s) can expand further. Biopsies and other minor thoracic operations do not decrease the ventilatory volume to the same extent as lobectomies. To sustain adequate exchange of gases in the ventilatory system, the autonomous nervous system may increase the ventilatory rate, but how surgery affects the ventilatory cycle is not well-known.

Because of the above-mentioned differences between the resective surgery and other minor thoracic operations, the allocation to intervention groups was done in these two blocks (resective and minor pulmonary surgery) separately. Nevertheless, the surgical technique was chosen by operative surgeon, and minor thoracic operations were more often done by VATS technique, whereas pulmonary resections were performed as likely either by thoracotomy or by VATS technique at the time of the study. This reflects also to these groups, and therefore, we decided to also compare intervention groups between the surgical techniques.

Based on these theoretical possible changes we intended to evaluate how breathing cycles change post-operatively, and how it may be improved by respiratory physiotherapy. IP offers new non-invasive tool to investigate this and the agreement of IP and PNT has been studied with tidal breathing parameters in adults and children [24, 25]. Good linearity of simultaneously recorded IP and PNT has already been shown in our thoracic and cardiac surgery patients both pre- and postoperatively [26].

We were able to detect alterations in respiration phase times, and that it could be influenced with two modes of physiotherapy. At POD1 after surgery respiratory phase times became more similar between intervention groups as a result of the surgery to respiration, but at POD2 difference between intervention groups arises. It seems that IMT physiotherapy may enhance the recovery of both inspiration and expiration of the breathing cycle, although there was no statistically significant difference during two first postoperative days. The increase in inspiration is easily explained by effect of inspiratory muscle physiotherapy, since inspiration normally is the active part of respiration, and this type of physiotherapy gives even more focus on active effort of inspiration. Expiration is passive in resting phase breathing but may also be enhanced by active effort. Our results indicate that IMT physiotherapy focusing on inspiration may also enhance expiration. In addition, breathing frequency seemed to be somewhat lower in the IMT group, but the difference between intervention groups was not statistically or clinically significant.

According to our results, it seems that IMT may have benefits especially among patients who were operated using thoracotomy, resulting a statistically significant difference between intervention groups in EI ratio. This could be explained by the more profound effect that thoracotomy incision had on the respiration and especially on inspiration, and therefore, physiotherapy modality focusing on active inspiration could be more beneficial to these patients.

Unfortunately, IP is not an independent measure of lung volumes nor intensity (volume per time), but it provides time-related functional factors that may further benefit clinicians when they evaluate the recovery of, e.g., high risk postoperative patients. Volume and intensity measurements would require calibration using volume spirometry multiple times to each patient separately, which was not possible in these surgical patients and, therefore, in this study design. As our experiences were favourable, the algorithms involved in this study may fairly easily be transferable to clinical monitoring.

The setting of our trial was a randomized clinical trial comparing two physiotherapy modalities in two blocks of surgeries, since the modality of surgery was often predetermined based on the patients’ preoperative condition and diagnosis. Our intervention groups were fairly comparable based on the background information, although some numeric differences in types of surgery were observed. However, these differences were not statistically significant, and although the volume resected could not be compared qualitatively, we compared in pairs both thoracotomy vs. VATS and major resections vs. minor resections but found no major differences in the main outcomes. Therefore, we assume the volume losses in both intervention groups likely were fairly comparable. In addition, a strength of this study is the relatively large number of patients in both blocks which gave us the opportunity to evaluate how the type of surgery (resective surgery or minor thoracic operation) and surgical technique (thoracotomy or VATS) may affect breathing cycle. Nevertheless, the heterogeneity of the study population sets limitations and may even lead to an underpowered study, even though our precalculated study power implied that the cohorts should be large enough even when assessed separately based on the extent of surgery.

Also, as a weakness in this study we acknowledge the short follow-up time of only two postoperative days, which was mainly determined by the length of stay at the surgical ward. With longer follow-up time it would have been possible to evaluate how the difference between intervention groups evolve after POD2. In addition, the monitoring and recording of impedance was not continuous throughout the day, but only for 10 min after physiotherapy session.

This study is first of a kind to evaluate breathing cycle and inspiration and expiration times by IP among postoperatiove patients, and therefore, there is no basis of comparison for what kind of chances in these times would be clinically relevant for patients’ recovery. We can assume, that the small differences detected in our follow-up would not have clinical relevance, but if the difference between intervention groups would continue to increase, that could lead to clinically relevant differences also.

## Conclusions

According to our results we can spot differences and changes in respiratory times using IP measurements among thoracic surgery patients. Physiotherapy overall is beneficial to patients’ recovery and it seems that IMT physiotherapy may have benefits over PEP physiotherapy among patients operated by thoracotomy, but more research is needed to evaluate how respiratory times progress after POD2, and how it correlates to patient’s recovery after first two postoperative days.

## Data Availability

Anonymous data may be used upon a reasonable request.

## Abbreviations

BrthFreqAvg: Average breathing frequency from a 10-min period
EI ratio: Ratio of expiration and inspiration times
IMT: Inspiratory muscle training physiotherapy
IP: Impedance pneumography
PEP: Water bottle positive expiratory pressure training physiotherapy
PNT: Pneumotachography
PPC: Postoperative pulmonary complication
POD1: Postoperative day one
POD2: Postoperative day two
Preop: Preoperative day
TexpAvg: Average expiration time in seconds from a 10-min period
TinspAvg: Average inspiration time in seconds from a 10-min period
VATS: Video assisted thoracic surgery
